# Proteomic Analysis Reveals that Proteasome Subunit Beta 6 Is Involved in Hypoxia-Induced Pulmonary Vascular Remodeling in Rats

**DOI:** 10.1371/journal.pone.0067942

**Published:** 2013-07-03

**Authors:** Jian Wang, Lei Xu, Xin Yun, Kai Yang, Dongjiang Liao, Lichun Tian, Haiyang Jiang, Wenju Lu

**Affiliations:** 1 Guangzhou Institute of Respiratory Diseases, State Key Laboratory of Respiratory Diseases, The First Affiliated Hospital of Guangzhou Medical University, Guangzhou Medical University, Guangzhou, Guangdong, China; 2 Division of Pulmonary and Critical Care Medicine, School of Medicine, Johns Hopkins University, Baltimore, Maryland, United States of America; 3 Department of Laboratory Medicine, The First Affiliated Hospital of Guangzhou Medical University, Guangzhou Medical University, Guangzhou, Guangdong, China; University of Pécs Medical School, Hungary

## Abstract

**Background:**

Chronic hypoxia (CH) is known to be one of the major causes of pulmonary hypertension (PH), which is characterized by sustained elevation of pulmonary vascular resistance resulting from vascular remodeling. In this study, we investigated whether the ubiquitin proteasome system (UPS) was involved in the mechanism of hypoxia-induced pulmonary vascular remodeling. We isolated the distal pulmonary artery (PA) from a previously defined chronic hypoxic pulmonary hypertension (CHPH) rat model, performed proteomic analyses in search of differentially expressed proteins belonging to the UPS, and subsequently identified their roles in arterial remodeling.

**Results:**

Twenty-two proteins were differently expressed between the CH and normoxic group. Among them, the expression of proteasome subunit beta (PSMB) 1 and PSMB6 increased after CH exposure. Given that PSMB1 is a well-known structural subunit and PSMB6 is a functional subunit, we sought to assess whether PSMB6 could be related to the multiple functional changes during the CHPH process. We confirmed the proteomic results by real-time PCR and Western blot. With the increase in quantity of the active subunit, proteasome activity in both cultured pulmonary artery smooth muscle cells (PASMCs) and isolated PA from the hypoxic group increased. An MTT assay revealed that the proteasome inhibitor MG132 was able to attenuate the hypoxia-induced proliferation of PASMC in a dose-dependent manner. Knockdown of PSMB6 using siRNA also prevented hypoxia-induced proliferation.

**Conclusion:**

The present study revealed the association between increased PSMB6 and CHPH. CH up-regulated proteasome activity and the proliferation of PASMCs, which may have been related to increased PSMB6 expression and the subsequently enhanced functional catalytic sites of the proteasome. These results suggested an essential role of the proteasome during CHPH development, a novel finding requiring further study.

## Introduction

Pulmonary hypertension (PH) is characterized by increased pulmonary artery pressure (PAP) and structural changes in the walls of pulmonary arteries (PA) causing pulmonary vascular remodeling. This disorder eventually leads to right ventricular failure and death. In addition to being a primary disorder, PH is also an accompanying complication of a variety of cardiopulmonary diseases, for example chronic obstructive pulmonary disease (COPD), which is highly prevalent and continues to be an increasing cause of morbidity and mortality worldwide [1], [2]. For patients with COPD, structural and functional changes in the PA induced by chronic hypoxia (CH) are associated with the development of PH. Despite numerous studies on the morphological and functional changes in PH in response to CH, the underlying cellular and molecular mechanisms remain unclear.

During the past decades, a series of proteins, including hypoxia-inducible factor (HIF) and bone morphogenetic protein receptor type II (BMPRII) have been shown to be significantly altered during the PH disease process in comparison with normal condition. These altered proteins are accompanied with changes in expression levels, and some of these changes have already been identified as participating factors in the remodeling of the pulmonary artery. These data suggest a crucial contribution of the changed levels of such proteins to the progression of PH disease. Protein quantity is controlled by both protein synthesis and protein degradation. The ubiquitin proteasome system (UPS), the major intracellular proteolytic system, is complex in both structure and function [3]. Degradation of proteins by the UPS occurs mainly in two steps: targeting of proteins with ubiquitin and successive degradation by the 26S proteasome. Enhanced proteasome activity is associated with proliferative diseases such as cancer [4]–[7] and cardiovascular disease [8]–[13]. The essential roles of the UPS during the degradation of cellular proteins, the regulation of the cell cycle, and the modulation of abnormal cellular proliferation make it an attractive target for the therapy of hyperproliferative chronic lung diseases such as lung cancer [14], [15] and pulmonary fibrosis [16]. Proliferation of PASMCs is a critical part of the pathogenesis of CHPH. However, to date, little is known about the role of the UPS during the development of PH. In consideration of the complex composition of the UPS, we sought to select proteins of interest using high throughput and sensitive methods such as proteomic analyses.

Proteomic analyses have been widely used to identify differentially expressed proteins, thus this technique is one of the most effective methods for delineating the relationship between the UPS and vascular remodeling during the CHPH process. The CHPH rat model used in this study is a commonly used model for studying pulmonary vascular remodeling [17], [18]. Furthermore, since proliferation of PASMCs is widely accepted as one of the main causes of vascular remodeling [19], we also focused on the medial layer of the distal PA, which is composed of PASMCs. Real-time PCR and Western blot analyses were applied to determine expression changes mRNA and protein levels, respectively. This study expands the knowledge of the mechanisms of development of CHPH by successfully demonstrating the involvement of a UPS functional subunit, PSMB6, in a CHPH rat model. Furthermore, this study uncovers the possibility of the potential contribution of the UPS system in the CHPH process, which to date, lacks sufficient data and requires further studies for better understanding of the disease process.

## Materials and Methods

### Exposure of Animals to Chronic Hypoxia

All procedures were approved by the Animal Care and Use Committee of Guangzhou Medical University. Twenty SD rats (male, 8 weeks, 0.25±0.03 kg) were provided by the Guangdong Laboratory Animal Centre, Guangzhou, China. These rats were randomized into two groups, the CH group and the control group. Rats in the CH group were placed in a hypoxic chamber for 21 days. As previously described [20]; the chamber was continuously flushed with a mixture of room air and N_2_ to maintain 10±0.5% O_2_ and CO_2_<0.5%. The O_2_ concentration in the chamber was continuously monitored using a PRO-OX unit (RCI Hudson, Anaheim, CA). Animals were exposed to room air for 10 minutes, once a week for cage cleaning and replenishment of food and water. Rats in the normoxia control group were housed in ambient room air next to the hypoxic chambers. The CHPH model was assessed by measuring the ratio of the right ventricle to left ventricle plus septum weight RV/(LV+S) and mean pulmonary artery pressure (mPAP).

### Preparation of Pulmonary Arteries

At the end of the hypoxic exposure, all rats were anesthetized with sodium pentobarbital (65 mg/kg i.p.). The hearts and lungs were removed en bloc and transferred to a petri dish containing a physiological salt solution (PSS; 130 mM NaCl, 5 mM KCl, 1.2 mM MgCl_2_, 1.5 mM CaCl_2_, 10 mM HEPES, and 10 mM glucose; the pH was adjusted to 7.2 with 5 M NaOH). Distal (4th generation) intrapulmonary arteries were dissected from the lungs, and adipose, adventitia, and connective tissues were carefully removed. The tissues were stored at −80°C prior to use. The arterial tissue was cut into pieces and placed in a glass homogenizer along with 200 µl of protein lysis buffer (7 M Urea, 2 M Thiourea,4% (w/v) CHAPS, 10 mg/ml DTT,2% Pharmalyte, and PMSF 20 mg/ml at pH 3–10). The tissue was carefully homogenized, and the solution was placed in a microcentrifuge tube. The samples were subjected to repeated freeze-thaw cycles by switching the protein lysate between liquid nitrogen and a 37°C water bath for three times. Then, the samples were centrifuged at 17000 rpm for 1 h. The supernatant was transferred into a new tube and the protein concentration was measured using the BCA Protein Assay Kit (BioRad, USA).

### Culture and Treatment of Rat Distal PASMCs

As previously described [21], the enzymatically isolated PASMCs were seeded in 35 mm culture dishes and cultured in Smooth Muscle Growth Medium-2 (SMGM-2, Clonetics, Walkersville, MD) in a humidified atmosphere of 5% CO_2_, 95% air at 37°C for 3–4 days. Cells were then growth-arrested in Smooth Muscle Basal Medium (SMBM, Clonetics, Walkersville, MD) containing 0.3% fetal bovine serum for 24 h before being exposed to normoxia or hypoxia (4% O_2_) in the presence or absence of MG132 for 60 h prior to various assessments. The O_2_ concentration in the incubator was continuously monitored by an O_2_ sensor (8–730 flow-through O_2_ microelectrodes, Microelectrodes Inc, Bedford, NH).

### Two-dimensional Electrophoresis (2DE) and Image Analysis

Each sample contained PA isolated from two rats and each group contained 10 rats, i.e., each group had 5 samples. Total amount of 190 µg protein from each sample was loaded onto immobilized pH gradient (IPG) gel strips (GE Healthcare, Biosciences, Uppsala, Sweden) and left overnight at room temperature. Each strip was overlaid with 2–3 ml of mineral oil to prevent evaporation during the rehydration process. The IPG strip was then placed on the tray, and isoelectric focusing was performed at 20°C using the IPG-Phor isoelectric system (BioRad, USA). The initial voltage was set at 250 V and raised stepwise to 4,000 V to remove the salt. The proteins were focused for 8 h at 80,000 V. After equilibration, the IPG strips were analyzed in 2D on a 12% (w/v) SDS polyacrylamide gel performed in a Protean II xi cell vertical electrophoresis tank (Bio-Rad,USA). Subsequently, the 2D gel was silver-stained and scanned. The digital images were analyzed using ImageMaster 2D Platinum 5.0 software (GE, USA) for spot-intensity calibration, spot detection, background abstraction, gel matching, and establishment of the average gel. The differentially expressed spots were defined as having a more than two-fold difference between the control and treated group. All data were statistically analyzed with SPSS13.0 software and Microsoft Excel 2003.

### Digestion of Proteins and Identification by Matrix-assisted Laser Desorption/ionization Time of Flight Mass Spectrometry

All five gels from each group were analyzed. The identified protein spots in the gel were excised from the gel and were in-gel digested. Briefly, the gel spots were destained in 30 mmol/L K_3_Fe(CN)_6_ and 100 mmol/L Na_2_S_2_O_3_, then dehydrated with 100% acetonitrile and dried in a stream of nitrogen gas. The dried gel pieces were incubated in a digestion solution consisting of 25 mM NH_4_HCO_3_ and 12.5 µg/ml trypsin (Promega, USA) for 16–18 h at 37°C. The tryptic peptide mixture was extracted and mixed with matrix a-cyano-4-hydroxycinnamic acid (CHCA) for mass spectrum analysis. Mass spectra results were obtained using an Applied Biosystems Voyager System 4800 matrix-assisted laser desorption/ionization time of flight mass spectrometry (MALDI-TOF) mass spectrometer (ABI, USA) with an accelerating voltage of MS/MS 8000 V. Mass fingerprinting was used for protein identification from tryptic fragment sizes in the NCBInr database (http://www.matrixscience.com) and SWISS-PROT database (http://web.expasy.org/docs/swiss-prot_guideline.html) with the MASCOT search engine for information such as protein name, mass score, and peptide match.

### Total RNA Extraction and Real-time PCR

Total RNA was extracted from the distal PA using the TRIzol reagent (Invitrogen, Carlsbad, CA). RNA quantification and purification were evaluated by measuring absorbance, and A260/A280 ratios in the range of 1.8–2.0 were considered satisfactory for purity standards. Denaturing agarose gel electrophoresis was used to assess the quality of the samples. Synthesis of cDNA was performed by reverse transcription from 1 µg total RNA, and mRNA was reverse transcribed using the iScript cDNA synthesis kit (BioRad). cDNA was quantified with real-time quantitative PCR (qPCR) in an iCyclerIQ machine (BioRad) using Quanti-Tect SYBR Green PCR Master Mix (Qiagen). The qPCR reaction mixture of 25 µl contained 200 nM each of the forward and reverse primers and a cDNA template from 6.25 ng of RNA. Primer sequences were as follows (5′-3′): PSMB6 F: (GACAAGCTGACCCCTATCCA; R: GCACTAGTGGAGGCTCGTTC), PSMB1 (F: TCTGCATCGTGACCAAAGAG; R: TAAAACAAACGTGCCACAGC), 18S (F: GCAATTATTCCCCATGAACG; R: GGCCTCACTAAACCATCCAA). The real-time qPCR program consisted of three steps, including a hot start at 95°C for 15 m, 40 cycles at 94°C for 15 s, 57.5°C for 20 s, and 72°C for 20 s, and melting curves were performed at 95°C for 1 min and 55°C for 1 min, with 80 repeats of incremental increases of 0.5°C. Detection of the cycle threshold (CT) values was performed with iCyclerIQ software. Specificity of the PCR products was confirmed by the melting curve and DNA sequencing. Standard curves were analyzed for all genes to assess the efficiency of amplification of each gene. The relative concentration of each transcript was calculated using the Phaffl method [22]. Data were expressed as a ratio of PSMB6 and PSMB1 to 18S in the same sample.

### Protein Isolation and Western Blotting

Pulmonary artery tissue was homogenized by sonication and dissolved in lysis buffer containing 62.5 mM Tris-HCl (pH 6.8), 2% sodium dodecyl sulfate (SDS), 10% glycerol, 5% protease inhibitor cocktail, 1 mM EDTA, and 200 µM 4-(2-Aminoethyl) benzenesulfonyl fluoride hydrochloride. Total protein concentration in the homogenates was determined using the BCA Protein Assay Kit (Bio-Rad, USA) with bovine serum albumin as a standard (Calbiochem, San Diego, CA). The extracted protein samples were stored at −80°C. Homogenates were denatured by adding dithiothreitol to 150 mM and heating at 95°C for 3 min. The homogenized proteins were resolved by 12% SDS-PAGE calibrated with prestained protein molecular weight markers (Precision Plus, Bio-Rad, Carlsbad, CA). Separated proteins were transferred to polyvinylidene difluoride membranes (pore size 0.45 µm, BioRad), and the membranes were blocked with 5% nonfat dry milk in Tris-buffered saline containing 0.2% Tween 20 and blotted with affinity-purified polyclonal antibodies specific for PSMB6 (C-16, Santa Cruz Biotechnology, USA), PSMB1 (11749-1-AP,Proteintech, USA), or a monoclonal antibody against α-actin (Sigma, USA). The membranes were then washed 3 times for 5 min each with a 1×Tris-buffered saline (TBS) solution with Tween-20 and incubated with horseradish peroxidase-conjugated rabbit anti-goat, goat anti-rabbit, or goat anti-mouse IgG for 1 h. Bound antibodies were detected using an enhanced chemiluminescence system (ECL, GE Healthcare, Piscataway, NJ).

### Proteasome Function Assays

Chymotryptic proteasome activities of PA and PASMCs were measured as previously described with a few minor modifications [23]. Briefly, PASMCs and PA were sonicated in homogenization buffer (25 mM Tris (pH 7.5), 100 mM NaCl, 5 mM ATP, 0.2% (v/v) Nonidet P-40, and 20% glycerol), and cell debris were removed by centrifugation at 4°C. Protein concentration in the resulting crude cellular extracts was determined using the BCA Protein Assay Kit. To measure 26S proteasome activity, 50 µg of protein from the crude cellular extracts of each sample was diluted with buffer I (50 mM Tris [pH 7.4], 2 mM dithiothreitol, 5 mM MgCl_2_, and 2 mM ATP) to a final volume of 200 µl (assayed in quadruplicate). The fluorogenic proteasome substrate, Suc-LLVY-AMC (chymotryptic substrate; Calbiochem), was dissolved in dimethyl sulfoxide (DMSO) to a final concentration of 80 mM. Proteolytic activities were continuously monitored for 2 h at 37°C by measuring the release of the fluorescent group, 7-amido-4-methylcoumarin (AMC), with a fluorescence plate reader (Vario Skanflas); the excitation and emission wavelengths were 380 and 460 nm, respectively.

### MTT Cell Proliferation Assay

PASMC proliferation was measured using an MTT assay (Sigma, USA). In short, PASMCs were seeded at a density of 5000 cells/cm^2^ in 96-well plates. The cells were incubated for 24 h in serum-free SMBM, and were then exposed to MG132 (0, 0.01, 0.05, 0.1, and 0.2 µM) for 60 h in normoxia or hypoxia (4% O_2_). Plain SMBM was used as the negative control. At the end of treatment, MTT at 0.25 mg/mL was added to the plates, and incubation continued for another 4 h at 37°C. The supernatant was then carefully removed, and 150 µl of DMSO was added to dissolve the formazan crystals. The absorbance of the solubilized product at 490 nm (A490) was measured with an ELISA reader (Thermo Scientific Varioskan Flash, USA). All determinations were confirmed in at least three identical experiments.

### PSMB6 Small Interfering RNA Transfection (siRNA)

The siRNA specific for PSMB6 (siPSMB6) was designed according to Genebank accession number NM_057099.3 for PSMB6 mRNA and was purchased as ON-TARGETplus SMARTpool from Thermo Fisher Scientific Inc. (Lafayette, CO). The detailed siRNA treatment protocol was performed per the manufacturer’s instructions. The effect of siPSMB6 was verified by Western blot. PASMCs at about 50∼60% confluence were transfected with 25 nM of non-targeting control siRNA (siNT) or siPSMB6 (using GeneSilencer [Genlantis, San Diego, CA]) for 6 h in serum-free SMBM. Serum was then added to a final concentration of 0.3% FBS. PASMCs were exposed to siRNA for 72 h before subsequent analysis with Western blot.

### 5′-bromo-2′-deoxyuridine (BrdU) Cell Proliferation Assay

The proliferation of PASMCs incubated with siPSMB6 was assessed using the Amersham Cell Proliferation Biotrak ELISA kit (GEhealthcare, Buckinghamshire, UK). Cells were plated into 96-well plates at 3 ×10 ^3^cells/well and cultured for 24 h in SMGM-2 basal medium. Then, the cells were transfected with 25 nM siRNA for 6 h in serum-free SMBM using GeneSilencer (Genlantis, San Diego, CA) according to the manufacturer’s instructions. After a further 36 h culture in SMBM containing 0.3% FBS, the cells were subjected to normoxic or hypoxic (4% O_2_, 60 h) exposure. The BrdU reagent was diluted (1∶100) and incorporated into the proliferating cells 24 h prior to subsequent ELISA assay for BrdU incorporation according to the manufacturer’s instructions. The amount of BrdU incorporated into the cells was measured as optical density (OD) using a spectrophotometer microplate reader (Benchmark microplate reader, BioRad, USA) at a wavelength of 450 nm.

### Statistical Analysis

Parameters are expressed as mean ± standard deviation. For the 2DE experiment, ‘n’ represents the number of samples; this is because we combined the PA from 2 rats as 1 sample. For the other experiments, ‘n’ is the number of experiments performed, which equals the number of animals providing PA or cells. Statistical comparisons were performed using a Student’s T-test or ANOVA as appropriate. Differences were considered to be significant when *P*<0.05.

## Results

### Verification of CHPH Model in Rats

Twenty male SD rats were randomly divided into two groups: normoxic group (10 rats) and CH group (10 rats). After the rats were placed in the CH chamber (10% O_2_) for 21 days, the CHPH model was successfully established as measured by the indexes of right ventricular systolic pressure (RVSP), mean pulmonary artery pressure (mPAP), and the weight ratio of right ventricle to left ventricle plus septum RV/(LV+S). Compared with the normoxic group, RVSP and mPAP were significantly increased in the CH group. Right ventricular hypertrophy also occurred in the CH group as indicated by the increased weight ratio of RV/(LV+S) (Table 1). These results are consistent with our previous report [24].

**Table 1:** Verification of the hypoxia-induced PAH model in rats in this study.

Group	mPAP (mmHg)	RVSP (mmHg)	RV/(LV+S)
Norm	11.6±0.52	24.2±0.08	0.26±0.02
Hypo	25.4±0.72*	52.3±1.67*	0.55±0.07 *

Norm: normal group n = 10, Hypo: Hypoxia group n = 10; RVSP: Right ventricular systolic pressure, mPAP mean pulmonary artery pressure, RV/(LV+S): right ventricle to left ventricle plus septum.

Table 1 illustrates the changes in the pressure indexes after SD rats were placed in a hypoxic atmosphere (10% O_2_, 21 days). *P<0.01 versus normoxia.

### Two-dimensional Electrophoresis Analysis of the Protein Expression in Distal PA Isolated from Rats Exposed to Normoxia and CH

**Figure 1:**
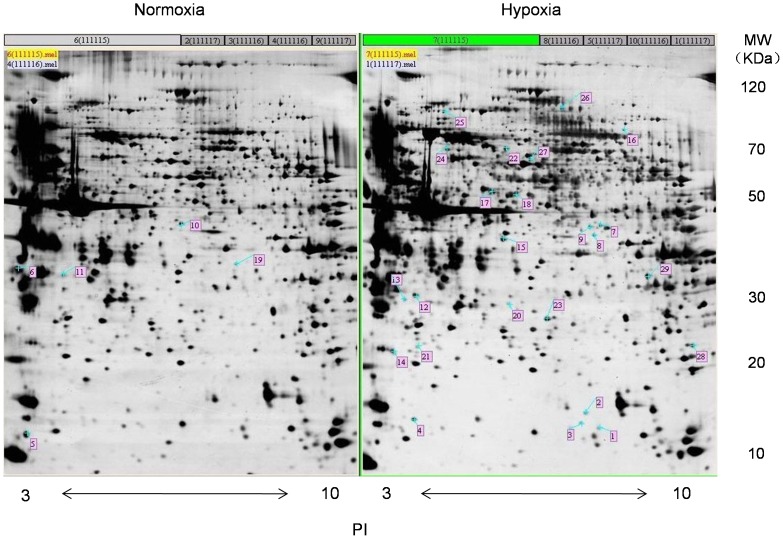
Two-dimensional electrophoresis maps. The two-dimensional electrophoresis maps represent the separation view of the extracted proteins in the distal PA isolated from normoxic and CHPH rats (10% O_2_, 21 days). PA whole protein (190 µg) was separated on y1y∼10 IPG strip in the first dimension and 12% SDS-PAGE in the second dimension. The up-regulated protein spots are marked in the gels. There are 5 spots in the normoxia group and 24 spots in the hypoxia group. The molecular markers are shown on the right of the gel. The PI information is shown on the bottom of both gels.

Protein samples of rat distal PA were pooled from both normoxic and CH groups and subjected to 2DE separation. Hundreds of highly-resolved protein spots were visible when the 2DE gels were silver-stained. The results of the quantitative comparison revealed that 29 protein spots were significantly differentially expressed between the CH group and the control group. The representative gels of the two groups are shown in Figure 1.

### MALDI-TOF MS Identified Differentially Expressed Proteins and Bioinformatics

The proteins with differential expression of at least 2-fold between the normoxic and CH group were considered to be differentially expressed. According to this criterion, the 29 spots marked in Figure 1 were selected for identification. In total, 22 out of the 29 spots were excised from the gels and identified with confidence by peptide mass fingerprinting and tandem mass spectrometry. The results showed that 17 proteins were up-regulated in the CH group while 5 proteins were up-regulated in the normoxic group. The detailed corresponding information of those differentially expressed proteins is listed in Table 2 and Table 3, among which PSMB1 and PSMB6 were identified as subunits of the proteasome within the UPS. Our study mainly focused on the differentially expressed key components of the UPS, and therefore, PSMB6 was further analyzed.

**Table 2:** Up-regulated proteins identified by MS or MS/MS and database searches in the hypoxia group.

Spot no.^a^	Protein description	Database	Accession no.^b^	Theoretical MW c/pI^d^	PI Score^e^	Intensity Mean±SEM		Ratio Hypoxia/Normoxia	*P* Value
						Normoxia	Hypoxia		
1	Transgelin	SwissProt	P31232|TAGL	22588.3/8.87	112	0.0248±0.1317	0.0069±0.0016	3.587	0.032
3	Phosphotriesterase related protein	NCBInr	61889077	39120/6.4	77	0.0079±0.0042	0.0024±0.0007	3.319	0.035
4	Hippocalcin like protein 1	SwissProt	P62749|HPCL1	22324/5.32	151	0.0365±0.0108	0.0192±0.0091	2.010	0.026
7	Branched chain specificacyl-CoA dehydrogenasemitochondrial	SwissProt	P70584|ACDSB	47793.7/8.27	141	0.0116±0.0049	0.0286±0.01534	2.461	0.045
8	Phosphotriesterase-related protein	SwissProt	Q63530|PTER	38906.9/6.06	52	0.0217±0.0079	0.0084±0.0034	2.585	0.008
9	Mannose-1 phosphateguanyltransferase beta	NCBInr	157817724	39876.6/6.27	84	0.0374±0.0108	0.0185±0.0045	2.023	0.305
12	Chloride intracellularchannel protein 1	SwissProt	Q6MG61|CLIC1	26963.8/5.09	333	0.0605±0.0099	0.3000±0.0073	2.0137	0.001
13	Inositol monophosphatase	SwissProt	P97697|IMPA1	30491.4/5.17	86	0.0218±0.0087	0.0106±0.0041	2.0575	0.037
14	Proteasome subunit beta type-6	SwissProt	P28073|PSB6	25273.4/4.85	77	0.0160±0.0017	0.0482±0.0059	3.011	0.003
15	Isocitrate dehydrogenase (NAD)subunit alpha, mitochondrial	SwissProt	Q99NA5|IDH3A	39588/6.47	184	0.0587±0.0099	0.0262±0.0138	2.235	0.003
17	Von Willebrand factor	SwissProt	Q642A6|VWA1	44804.3/6.22	108	0.0755±0.02643	0.0174±0.0070	4.330	0.004
18	Tubulin gamma1 chain	SwissProt	P83888|TBG1	51068.7/5.66	53	0.0558±0.01365	0.0272±0.0594	2.047	0.006
23	Endoplasmic reticulumprotein ERp29	SwissProt	P52555|ERP29	28556.9/6.23	170	0.0774±0.01865	0.02077±0.0100	3.727	0.001
25	Ribosome binding protein 1	NCBInr	293358103	144481.3/9.05	141	0.0076±0.0019	0.01963±0.0066	2.5510	0.026
26	Procollagen-lysine,2-oxoglutarate 5-dioxygenase 2	SwissProt	Q811A3|PLOD	84488.5/6.26	116	0.0085±0.0008	0.6784±0.0525	7.9594	0.035
27	Dihydropyrimidinase related protein 2	SwissProt	P47942|DPYL2	62238.6/5.95	137	0.0027±0.0016	0.01376±0.0046	5.0718	0.004
28	Proteasome subunit beta type-1	SwissProt	P18421|PSB1	26462.3/6.9	73	0.0132±0.0021	0.0477±0.0154	3.602	0.001

a Protein spot numbers on 2DE gel.

b Accession numbers from NCBI database (accessible at http://www.ncbi.nlm.nih.gov/) or the Swiss-Prot Protein Database (http://web.expasy.org/docs/swiss-prot_guideline.html).

c Theoretical molecular mass.

d Theoretical PI.

e MASCOT protein score (based on combined MS and MS/MS spectra) of greater than 64 (*P*<0.05) or the total ion score (based on MS/MS spectra) of greater than 30 (*P*<0.05).

**Table 3:** Up-regulated proteins identified by MS or MS/MS and database searches in the normoxia group.

Spot no.^a^	Protein description	Database	Accession no.^b^	TheoreticalMW c/pI^d^	PI Score e	IntensityMean±SEM		Ratio Normoxia/Hypoxia	*P* Value
						Normoxia	Hypoxia		
5	Cytochrome-b5	SwissProt	sp|P00173|CYB5	15345.6/4.9	180	0.1007±0.0161	0.0472±0.0206	2.133	0.002
6	Polymerase I and transcript release factor	SwissProt	sp|P85125|PTRF_	43882/5.34	89	0.4068±0.1572	0.1916±0.0416	2.122	0.018
10	NADH dehydrogenase1 alpha subcomplex subunit 10	SwissProt	sp|Q561S0|NDUA	40467.6/7.64	176	0.0493±0.0146	0.0199±0.0096	2.472	0.008
11	Adiponectin	NCBInr	gi|21426809	26392.9/5.42	133	0.0550±0.0162	0.0187±0.0051	2.938	0.001
19	Sulfotransferase 1A1	SwissProt	sp|P17988|ST1A1	33884/6.37	117	0.0300±0.0137	0.0099±0.0031	3.0065	0.0257

a Protein spot numbers on 2DE gel.

b Accession numbers from NCBI database (accessible at http://www.ncbi.nlm.nih.gov/) or the Swiss-Prot Protein Database (http://web.expasy.org/docs/swiss-prot_guideline.html).

c Theoretical molecular mass.

d Theoretical PI.

e MASCOT protein score (based on combined MS and MS/MS spectra) of greater than 64 (*P*<0.05) or the total ion score (based on MS/MS spectra) of greater than 30 (*P*<0.05).

### Real-time PCR Analysis of the mRNA Expression of PSMB6 and PSMB1

**Figure 2:**
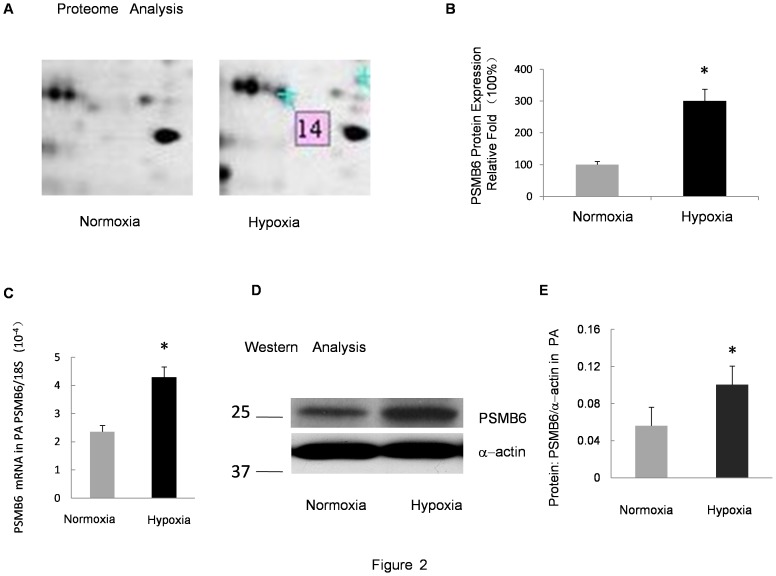
Verification of PSMB6 expression in the PA under prolonged hypoxia. **A:** Zoomed-in view of protein spot 14 in the 2DE gel in Fig. 1. **B:** Relative protein expression of PSMB6 in the 2DE gel. The normoxia group was considered as 100% and the relative fold-change in protein level is shown by spot density. **C:** Relative mRNA levels for PSMB6 from the PA of rats exposed to 10% O_2_ for 21 days compared with the normoxia control. Bar values are mean ± SEM (n = 5 in each group). *P<0.05 versus respective normoxia control. **D:** Protein levels of PSMB6 measured by Western blot in the PA isolated from rats exposed to hypoxia (10% O_2_, 21 days) or normoxia. **E:** Band intensity of PSMB6 normalized to α-actin. Mean values ± SEM were calculated from three independent samples. *P<0.01 versus normoxia.

**Figure 3:**
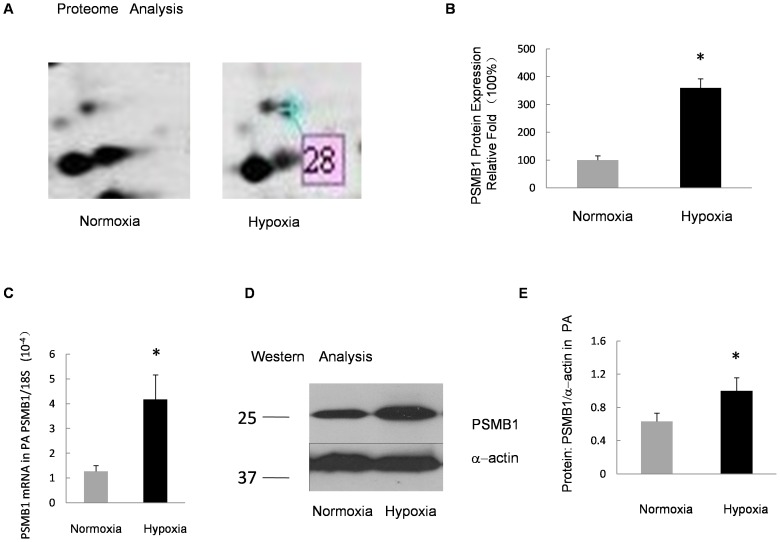
Verification of PSMB1 expression in PA under prolonged hypoxia. **A:** Zoomed-in view of protein spot 28 in the 2DE gel in Fig. 1. **B:** Relative protein expression of PSMB1 in the 2DE gel. The normoxia group was considered as 100% and the relative fold-change in protein level is shown by spot density. **C:** Relative mRNA levels for PSMB1 from the PA of rats exposed to 10% O_2_ for 21 days compared with normoxia control. Bar values are mean ± SEM (n = 5 in each group). *P<0.05 versus respective normoxia control. **D:** Protein levels of PSMB1 measured by Western blot in PA isolated from rats exposed to hypoxia (10% O_2_, 21 days) or normoxia. **E:** Band intensity of PSMB1 normalized to α-actin. Mean values ± SE were calculated from three independent samples. *P<0.01 versus normoxia.

In an attempt to confirm the proteomic results, alterations in the mRNA level of PSMB6 in the distal PA in CH group and the control group were analyzed using real-time PCR. As shown in Figure 2C and Figure 3C, PSMB6 and PSMB1 mRNA levels were up-regulated in the isolated distal PA from CH rats as compared with the normoxia controls.

### Western Blotting Confirmed the Up-regulation of PSMB6 and PSMB1

The increased expression of PSMB6 and PSMB1 was validated through Western blotting. Figure 2 shows the comparison of PSMB6 and α-actin in the distal PA isolated from the control group and the CH group. Figure 3 shows the results of the comparison between PSMB1 and α-actin. Results indicated that the expression of PSMB6 and PSMB1 in the CH group was significantly higher than that of the normoxic control group (*P*<0.01). These data were in accordance with the results obtained through the 2DE.

### Identification of Proteasome Activities in PASMCs and PA Isolated from Rats Exposed to both Normoxia and Hypoxia

**Figure 4:**
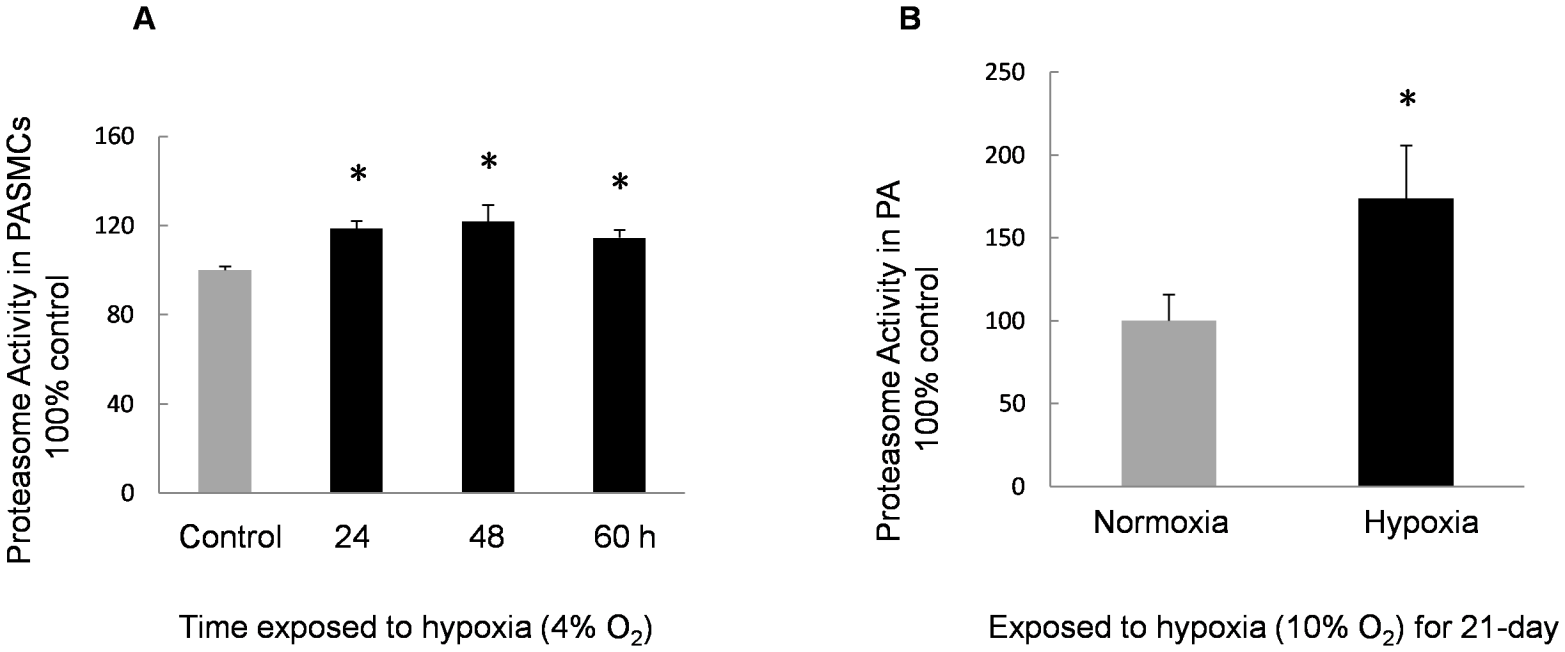
Proteasome activity of PASMCs and PA exposed to hypoxia. **A:** Activity of 20S proteasome in PASMCs incubated with normoxia or hypoxia (4% O_2_, 60 h). The activity in the control group was considered as 100%. Proteasome activities in PASMCs exposed to hypoxia were enhanced compared with the normal controls (n = 4, *P<0.05). **B:** Activity of 20S proteasome in PA isolated from normoxic or hypoxic (10% O_2_, 21 days) rats. The activity in the normoxic control group was considered as 100% and the proteasome activity in hypoxic group was approximately 1.7-fold of the normal control (n = 3, *P<0.05).

We analyzed the proteasome activity in hypoxia-exposed PASMCs *ex vivo* and in the distal PA from CHPH rats *in vivo.* The chymotrypsin-like activity of PASMCs and PA from normoxic and hypoxic conditions was measured. Endpoint analysis (120 min) of the 20S proteasome activity from the hypoxic group is shown as percent to normoxic control, while the activity of the 20S proteasome in the control was considered to be 100%. Results demonstrated that the proteasome activity in hypoxia-exposed PASMCs was significantly enhanced compared with the normoxic controls (Figure 4). In the distal PA from rats exposed to CH for 21 days, the proteasome activity was observed to be approximately 70% higher than the normoxic control.

### Analysis of PASMC Proliferation by MTT Assay

**Figure 5:**
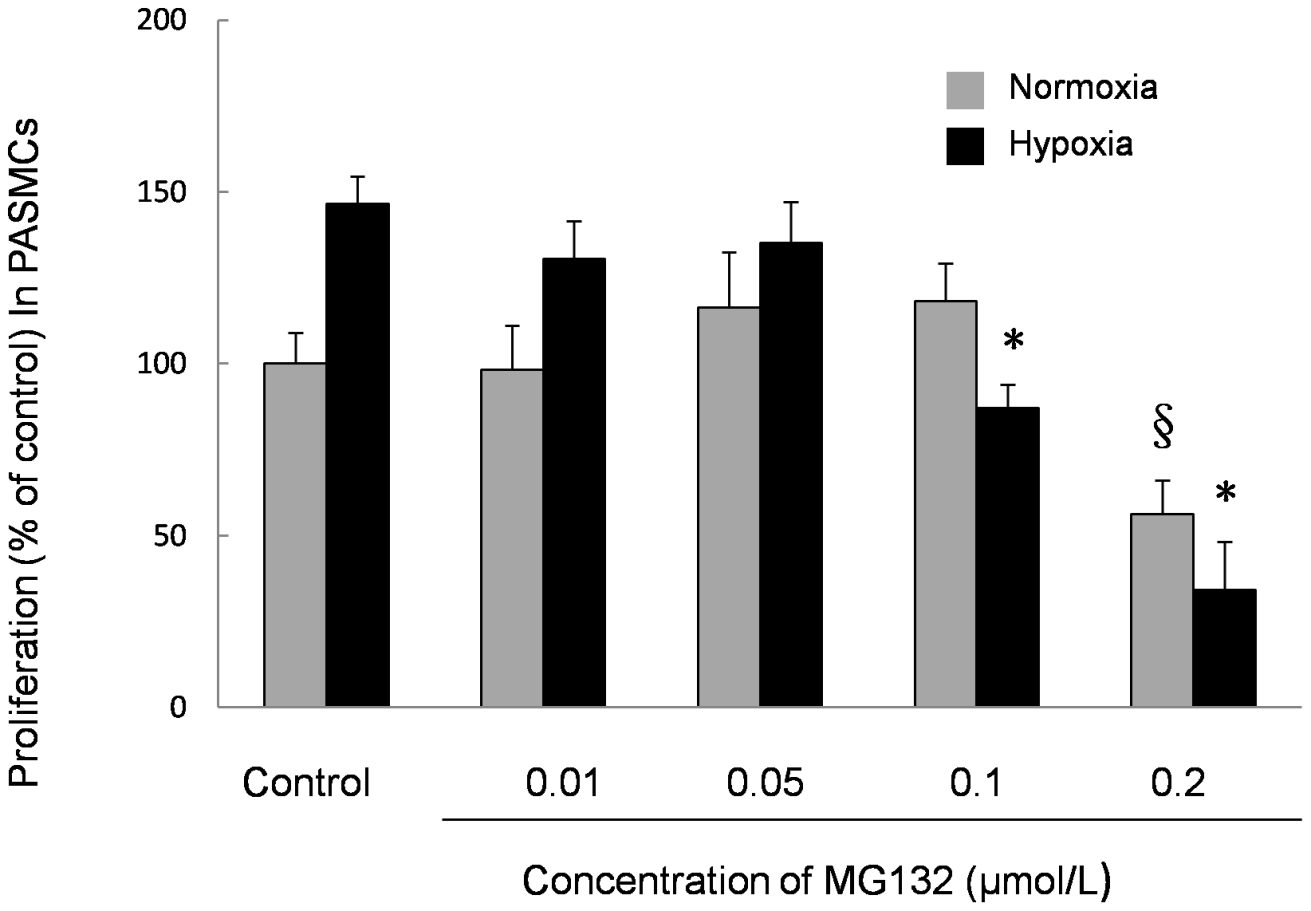
Effect of MG132 on proliferation of PASMCs induced by prolonged hypoxia. PASMCs were treated with normoxia or prolonged hypoxia (4% O_2_, 60 h) with or without MG132 at various dosages (0.01 to 0.2 µM). The bar graph represents the effects of prolonged hypoxia and MG132 on the proliferation of PASMCs by MTT assay. Bar values are means ± SEM. *P<0.05 compared with normoxia control. §*P*<0.05 compared with hypoxic control (n = 3 in each group).

To investigate the role of the proteasome in vascular remolding during rat CHPH development, an MTT assay was performed. PASMCs were seeded in a 96-well plate at a density of 5×10^3^ cells per well and were exposed to various concentrations of MG132, a proteasome inhibitor, for 60 h under either normoxic or hypoxic conditions. Results revealed that hypoxia could induce the proliferation of PASMCs, while MG132 treatment remarkably attenuated such hypoxic induction in a dose-dependent manner (Figure 5).

### Knockdown of PSMB6 Prevented Hypoxia-induced Proliferation

**Figure 6:**
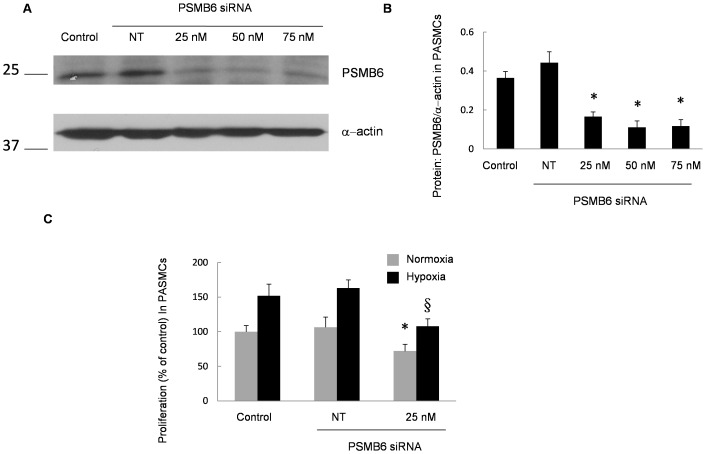
Effects of PSMB6 siRNA on the proliferation of PASMCs induced by prolonged hypoxia. **A** and **B**: The effects of PSMB6 siRNA (siPSMB6) on PSMB6 protein expression in PASMCs. PASMCs were transfected with 25 nM of non-targeting control siRNA (siNT) or siPSMB6 for 6 h in serum-free SMBM. PASMCs were then exposed to siRNA for 72 h before subsequent analysis with Western blotting. **C**: Effects of prolonged hypoxia and siPSMB6 on the proliferation of PASMCs by BrdU assay. PASMCs were treated with normoxia or prolonged hypoxia (4% O_2_, 60 h) with or without siPSMB6 treatment at dosages of 25 nM. Bar values are means ± SEM. *P<0.05 compared with normoxia nontargeting siRNA. §*P*<0.05 compared with hypoxic nontargeting siRNA (*n* = 3 in each group).

The effects of PSMB6 inhibition by siRNA on proliferation were also evaluated. Indeed, by using siPSMB6 at a dose of 25 nM, an approximate 60% decrease in the expression of PSMB6 proteins was observed (Figures 6A and B). To estimate the functional influences of PSMB6, we further investigated the effects of siPSMB6 on PASMC proliferation. As shown in Figure 6C, exposure to prolonged hypoxia (4% O_2_, 60 h) lead to a 51% increase in PASMC proliferation; treatment with siPSMB6 inhibited these increases.

## Discussion

In this study, we employed a classical proteomic strategy, 2DE for protein separation and mass spectrometry, to investigate differentially expressed proteins in the distal PA isolated from rats exposed to CH and normoxia. An important component of the UPS, PSMB6, was initially detected to be significantly up-regulated in the distal PA from CH rats. This up-regulation was further confirmed by real-time PCR and Western blotting. Additionally, proteasome activity was also enhanced by hypoxia in both PA and cultured PASMCs. Both of the proteasome inhibitor, MG132, and siPSMB6 could ameliorate hypoxia-induced proliferation in PASMCs. These results suggest that increased PSMB6 and subsequently enhanced proteasome activity may be involved in hypoxia-induced pulmonary vascular remodeling during the development of CHPH.

Exposure to CH can lead to PH both in rats [24] and mice [25], and these models have widely been accepted in the study of the mechanisms of CHPH. In our current study, a CHPH rat model was successfully established as verified by measuring mPAP and the ratio of RV/(LV+S).

UPS is one of the major cellular regulatory mechanisms involved in control of protein quality, the cell cycle, transcriptional factor regulation, gene expression, and cell differentiation [3], [26]. With its crucial role in the pathogenesis of many diseases associated with cell proliferation, such as cancer and cardiovascular disease, we hypothesized that UPS could possibly participate in vascular remodeling during CHPH in either a direct or indirect way. However, composed of thousands of proteins, the UPS is a very complex system. Thus far, little is known about the relationship between UPS and PH. Therefore, it is important and meaningful to find any clue to this complex puzzle using proteomic approaches.

By 2DE, imaging analysis, mass spectrometry, and MALDI-TOF MS, 22 differentially expressed proteins with different cellular functions were revealed. Among them, two proteins, PSMB1 and PSMB6, are proteasome subunits. The proteasome is responsible for the degradation of abnormal or damaged proteins, and therefore plays an important role in quality control. The 26S proteasome is a large complex consisting at least 66 subunits. PSMB5, 6, and 7 are the main catalytic sites associated with proteasome activity [27]–[30]. Because PSMB1 is a structural subunit [31], we primarily focused on PSMB6. To confirm the results of our proteomic analysis, we subsequently verified that PSMB6 and PSMB1 RNA and protein expression increased in rat CHPH models compared with normoxic control by real-time PCR and Western blot.

Since PSMB6 functions as the catalytic site of the proteasome, proteasome activity is likely to be enhanced as a result of its up-regulation, and such enhancement might be associated with the development of CHPH. Therefore, we analyzed whether hypoxia-related increases in PSMB6 could enhance the proteasome activity *in vivo* and *ex vivo*. Hypoxia-induced PASMC proliferation is well accepted as an important contributor to the progression of pulmonary hypertension [32], [33]. **S**ince the enhancement of proteasome activity is related to cell proliferation, we treated PASMCs with MG132, a commonly used proteasome inhibitor with potent, reversible, and cell-permeable characteristics. After exposure to various concentrations (0, 0.01, 0.05, 0.1, and 0.2 µM) of MG132 for 60 h under normoxia and prolonged hypoxic conditions, the hypoxia-induced PASMC proliferation was significantly inhibited at 0.1 and 0.2 µM, indicating that the proteasome may be involved in pulmonary artery remodeling.

To clarify whether PSMB6 expression was related to vascular remodeling, we used specific siRNA to knock down the expression of PSMB6 and found that decreased PSMB6 expression significantly normalized proliferation induced by prolonged hypoxia (4% O_2_, 60 h). These results indicate that increased PSMB6 expression in PASMCs exposed to hypoxia is associated with vascular remodeling.

These findings were largely consistent with earlier studies in other animal models, indicating the role of proteasome activity in cardiovascular disease and demonstrating that the use of a proteasome inhibitor could rescue vascular or heart remodeling and suppress further progression of hypertrophy or proliferation [10], [34]–[36]. All these findings suggest that increased PSMB6 expression was involved in the pathogenesis of rat CHPH.

The finding that proteasome is involved in pathogenesis of PH may provide a new strategy for its treatment. Kim et al. found that a proteasome inhibitor ameliorated PA in chronic hypoxia- and monocrotaline-induced PH animal models [37]. Some transcriptionsl factors associated with hypoxia diseases, like COPD and pulmonary hypertension, are also related to the proteasome. The Nrf2 activator oltipraz, which was demonstrated to attenuate CHPH in mice [38], can regulate PSMB6 expression [39]. TCF11, which plays an important role in COPD, is a key regulator for UPS [40] and 26S PSMB6 [41].

In our experiments, increased PSMB6 expression and proteasome activity in a hypoxia-induced animal model are significantly more pronounced in PA than those in cultured PASMCs exposed to hypoxia. This may indicate that other cell types are involved. Since adventitia and connective tissue were carefully removed, endothelial cells may be required for the observed effects. Kim et al. found that bortezomib, a proteasome inhibitor, could increase eNOS and NO expression in PA endothelial cells both *in vivo* and *in vitro* [37]. It was also reported that MG132 could inhibit the growth of calf pulmonary arterial endothelial cells and also induce apoptosis [42]. It is plausible that PSMB6 may also play a role in endothelial cells; however, further study is required to elucidate the mechanism(s).

2DE, in combination with MS, plays a central role in proteomics; however, several limitations and issues have been recognized [43]. The most important drawback of 2DE-based proteomics is that it is not possible to analyze the entire proteome. Low-abundant proteins, which include regulatory proteins, receptors, and other proteins that play key roles in cellular processes, are not detected by conventional staining. Silver staining detects the quantity of protein in a silver-stained spot is generally low [44]. This may be the reason why prominent markers of hypoxia, such as HIF-1, were not observed.

In summary, the present study revealed for the first time that one of the proteasome subunits, PSMB6, was increased in the distal PA from a CHPH rat model. The proteasome activity and the proliferation of PASMCs were also found to be up-regulated. However, both the proteasome inhibitor MG132 and siPSMB6 could prevent hypoxia-induced proliferation in PASMCs. These results suggest an association between increased PSMB6 and the development of CHPH, as well as the likely essential role of PSMB6 during the CHPH process. However, the detailed mechanism remains largely unclear and requires further exploration.
